# A new endemic pearl cichlid of the ‘*Geophagus*’ *brasiliensis* (Cichliformes: Cichlidae) species group from the Piranga River, upper Doce River basin, southeastern Brazil

**DOI:** 10.1111/jfb.70324

**Published:** 2026-01-05

**Authors:** Cidimar E. de Assis, Jorge A. Dergam, Amanda F. Cunha, Valéria N. Machado, Tomas Hrbek, Natállia M. de F. Vicente, Victor de Queiroz, Elisabeth Henschel

**Affiliations:** ^1^ Laboratório de Sistemática Molecular e Biologia da Reprodução (Beagle), Departamento de Biologia Animal Universidade Federal de Viçosa Viçosa Minas Gerais Brazil; ^2^ Laboratório de Evolução de Invertebrados Aquáticos (LEIA), Departamento de Biologia Animal Universidade Federal de Viçosa Viçosa Minas Gerais Brazil; ^3^ Laboratório de Evolução e Genética Animal (LEGAL), Departamento de Genética Universidade Federal do Amazonas Manaus Amazonas Brazil

**Keywords:** Atlantic forest, fish taxonomy, freshwater fish, Geophagini, integrative taxonomy, mitochondrial DNA

## Abstract

A new species of pearl cichlid of the ‘*Geophagus*’ *brasiliensis* species group, endemic to the Piranga River, a major tributary of the upper Doce River basin in the state of Minas Gerais, southeastern Brazil, is herein described. The new species is delimited using an integrative approach, with molecular‐based species delimitation methods coupled with morphological diagnosis. For this, we developed a matrix containing 27 sequences of the mitochondrial gene cytochrome c oxidase subunit I (*COI*) of species of the ‘*G*.’ *brasiliensis* species group obtained from GenBank and three new sequences generated in this work. The species delimitation method applied to the morphological characters was Population Aggregation Analysis (PAA), and the species delimitation methods applied to the nucleotide sequences were the branch coalescence methods: Bayesian Poisson Tree Processes (bPTP), General Mixed Yule Coalescent (GMYC) single‐threshold and GMYC multi‐threshold. The three molecular‐based species delimitation methods corroborate that the haplotypes of ‘*Geophagus*’ from the Piranga River represent a new species distinguished from all other in the ‘*G*.’ *brasiliensis* species group by exclusive presence of clear, rounded spots in the posteriormost dorsal‐fin rays in fixed adult specimens, and the combination of the following morphological characters: long snout (46.22%–52.74% of head length), tall head (94.08%–101.15% of head length), terminal mouth, 25 or 26 longitudinal scales on the E0 series, 14 or 15 spines on the dorsal fin, absence of bluish dots on the most anterior soft rays of the anal fin in live specimens and absence of bluish and longitudinal lines in the basal region of the caudal fin of live specimens. This work is the first description of a new species of the ‘*G*.’ *brasiliensis* species group from the Doce River basin.

## 1 INTRODUCTION

The Doce River basin is located in the state of Minas Gerais and Espírito Santo, southeastern Brazil, and is one of the major components of the Southeast Atlantic hydrographic region. The Doce River begins at the confluence of the Piranga and Carmo rivers in the municipality of Rio Doce in Minas Gerais (ANA, 2016) and is divided into three informal subdivisions, according to Vieira (2009): the upper Doce River, comprising the drainages and tributaries located upstream of the mouth of the Matipó River; the middle Doce River, between the mouth of the Matipó River and the border between Minas Gerais and Espírito Santo; and the lower Doce River, from this point to its mouth in the Atlantic Ocean, in Regência Augusta, district of Linhares, in Espírito Santo. The main channel of the Doce River, the Gualaxo do Norte and the Carmo rivers were directly impacted by the spill of iron‐ore tailings after the collapse of the Fundão Dam (owned by the mining company Samarco) in Bento Rodrigues, district of Mariana in Minas Gerais, in November 2015 (Salinas, 2016; Wanderley et al., 2016). This environmental disaster caused serious damage to fauna and flora, including fish kills and habitat degradation along the river channel (Espindola et al., 2016). The Piranga River sub‐basin is located in the upper Doce River section (Vieira, 2009) and was not directly impacted by the passage of mining tailings from the Fundão Dam (Vergilio et al., 2021). However, it has been affected by the discharge of domestic sewage, heavy metals and agricultural runoff activities (Fraga et al., 2021). This river includes some of the last known localities where the critically endangered catfish *Steindachneridion doceanum* (Eigenmann & Eigenmann, 1889), popularly known as ‘Surubim‐do‐doce’, still occurs (Drummond et al., 2021; Garavello, 2005). The Piranga River sub‐basin has been recognized as one of the areas of endemism within the Doce River basin (Sarmento‐Soares et al., 2022) and may represent one of the most important tributaries for repopulation and recovery of fish diversity in the main channel of the Doce River. However, knowledge of its ichthyofauna is scarce, and no comprehensive survey studies have been carried out to date.

Cichlidae is the most diverse family within the order Cichliformes, encompassing 258 valid genera and 1765 species distributed across tropical and subtropical regions of the Americas, Africa and southern Asia (Fricke et al., 2025). The Neotropical genus *Geophagus* Heckel, 1840, comprises 33 species (Fricke et al., 2025), popularly known as ‘South American Eartheaters’ and ‘Pearl Cichlids’. These fishes are highly appreciated in the ornamental aquarium trade for their bright body and fin colours, which may include bluish, greenish, yellowish or reddish, generally accompanied by elongated fin rays (Kullander, 1986; Weidner, 2000). Species of *Geophagus* are recognized by the following combination of morphological characters: the presence of a fleshy lobe on the upper branch of the first branchial arch, the presence of supraneural bone and scales on the dorsal fin and eyes closer to the dorsal profile of the head (Gosse, 1975; Heckel, 1840; Kullander, 1986). Currently, species of *Geophagus* are allocated into three phylogenetic groups (Kullander, 1986, 1998; López‐Fernández et al., 2005,b): the *Geophagus* sensu stricto species group, distributed across the Amazon, Orinoco, Tocantins‐Araguaia and Parnaíba River basins; the *Geophagus steindachneri* species group, found in the trans‐Andean basins between southern Panama to Lake Maracaibo in Venezuela; and the ‘*Geophagus*’ *brasiliensis* species group, restricted to river basins in eastern South America. According to recent phylogenetic studies, these three groups together do not constitute a monophyletic lineage (Chuctaya et al., 2022; Ilves et al., 2018; López‐Fernández et al., 2010). Consequently, when describing new species belonging to the last two groups mentioned above, the authors have provisionally assigned them to the genus ‘*Geophagus*’, and apostrophes between the genus name indicate the uncertain phylogenetic placement of these taxa (Mattos & Costa, 2018).

The ‘*G*.’ *brasiliensis* species group is represented by eight species that are widely distributed in rivers and lakes in southern, southeastern and northeastern Brazil, as well as eastern Uruguay (Argolo et al., 2020; Vieira‐Guimarães et al., 2024). These species are ‘*G*.’ *brasiliensis* (Quoy & Gaimard, 1824) from streams of Guanabara Bay in the municipality of Rio de Janeiro; ‘*Geophagus*’ *diamantinensis* Mattos, Costa & Santos, 2015, from the upper Paraguaçu River in the Chapada Diamantina region, Bahia; ‘*Geophagus*’ *iporangensis* Haseman, 1911, from the Ribeira do Iguape River basin in the state of São Paulo; ‘*Geophagus*’ *itapicuruensis* Haseman, 1911 from the Itapicuru River basin, northern Bahia; ‘*Geophagus*’ *multiocellus* Mattos & Costa, 2018 known from the Contas River basin, Bahia; ‘*Geophagus*’ *obscurus* (Castelnau, 1855) from the Paraguaçu River basin, Bahia; ‘*Geophagus*’ *rufomarginatus* Mattos & Costa, 2018, from the Buranhém River basin, Bahia; and ‘*Geophagus*’ *santosi* Mattos & Costa, 2018, from the Mariana River basin, Bahia. The nominal species ‘*G*.’ *brasiliensis* is still considered a widely distributed species across several coastal rivers in eastern Brazil, for example, the Paraíba do Sul, Mucuri, Jucuruçu and Jequitinhonha rivers (Mattos et al., 2015). In the Doce River basin, the first taxonomic record for this group was conducted by Haseman (1911), who analysed some specimens from the basin and identified them as ‘*G*.’ *brasiliensis*. More than a 100 years later, Argolo et al. (2020) discussed the occurrence of ‘*G*.’ *rufomarginatus* in the tributaries of the upper and middle Doce River, and the geographic distribution of this species expanded to the coastal basins between the Buranhém and Paraíba do Sul rivers, including the Doce River. However, the identity of ‘*G*.’ *brasiliensis* populations in the Doce River basin remains uncertain and controversial at the species level. As a result, specimens from this basin have been provisionally identified as ‘*G*.’ aff. *brasiliensis* or even as ‘*G*.’ cf. *brasiliensis* in more recent studies on regions of endemism in the Atlantic Forest (Vieira‐Guimarães et al., 2024). These uncertainties underscore the need for further taxonomic research in the Doce River basin, as some populations currently identified as ‘*G*.’ *brasiliensis* may in fact represent undescribed species.

The present study describes a new species of the ‘*G*.’ *brasiliensis* species group endemic to the Piranga River, a tributary of the upper Doce River in the state of Minas Gerais. We adopted an integrative approach, as proposed by Dayrat (2005), to describe this new taxon, combining morphological and molecular evidence with four species delimitation methods based on nucleotide sequences and morphological characters.

## 2 MATERIALS AND METHODS

### 2.1 Ethical statement and specimen preservation

Specimens were collected with permit number 14975–1, granted by the Sistema de Autorização e Informação em Biodiversidade, Instituto Chico Mendes de Conservação da Biodiversidade (ICMBio, Brazil). These specimens were euthanized in eugenol solution (Ross & Ross, 2008), and then fragments of muscle tissue were removed and stored in 96% alcohol for molecular analysis, according to the Animal Use Ethics Committee of Federal University of Viçosa (UFV), number 2040523303. After this procedure, individuals were fixed in 10% formaldehyde solution for 7 days, subsequently preserved in 70% ethanol (Auricchio & Salomão, 2002) and finally deposited in the ichthyology collection of the Zoology Museum of Federal University of Viçosa (MZUFV).

### 2.2 Morphological analyses

The material examined is deposited in the following ichthyological collections: MZUFV, João Moojen Zoology Museum, Federal University of Viçosa, Viçosa, Minas Gerais, Brazil; MNHN, National Museum of Natural History, Paris, France; FMNH, Field Museum of Natural History, Chicago, IL, USA; UFRJ, Biology Institute, Federal University of Rio de Janeiro, Rio de Janeiro, Rio de Janeiro, Brazil. Type material of ‘*G*.’ *brasiliensis*, ‘*G*.’ *diamantinensis*, ‘*G*.’ *iporangensis*, ‘*G*.’ *itapicuruensis*, ‘*G*.’ *multiocellus*, ‘*G*.’ *obscurus*, ‘*G*.’ *rufomarginatus* and ‘*G*.’ *santosi* was examined based on the information available in the taxonomic literature and photographs available online. Measurements and meristic counts follow Gosse (1975), Kullander (1986), Mattos et al. (2015) and Mattos and Costa (2018). Measurements are presented as percentages of standard length (SL), except for the head subunit measurements, which are presented as percentages of head length (HL). Measurements and counts were made on the right side of the specimens, whenever possible. Osteological preparations followed the protocol of Taylor and Van Dyke (1985); osteological nomenclature follows Costa (2006) and Mattos and Costa (2018). The software ImageJ version 1.54 g (Schneider et al., 2012) was used to obtain measurements of snout length from photographs of the holotype of ‘*G*.’ *brasiliensis*.

### 2.3 DNA extraction, amplification and sequencing

Tissue samples from three specimens from the Piranga River are stored at −20°C in the Laboratory of Molecular Systematics and Reproductive Biology, Department of Animal Biology, Federal University of Viçosa. Total genomic DNA was extracted following the CTAB 2% (cetyltrimethylammonium bromide) protocol (Doyle & Doyle, 1987). A fragment of approximately 650 base pairs of mitochondrial gene cytochrome c oxidase subunit I (*COI*) was amplified using the primers FishF2 and FishR1 (Ivanova et al., 2007). The 15 μL polymerase chain reaction (PCR) mix included 1.2 μL of 10 mM dNTPs (2.5 mM each DNTP), 1.5 μL 10× buffer [75 mM Tris HCL, 50 mM KCL, 20 mM [NH_4_]_2_SO_4_], 1.2 μL 25 mM MgCl_2_, 1.5 μL of each primer (2 pmol each), 0.5 μL of Taq DNA polymerase, 1 μL of template DNA and 6.6 μL ddH_2_O. PCR conditions were as follows: 94°C (30 s); 35 cycles of 94°C (30 s), 50°C (35 s), 72°C (90 s); followed by 72°C (5 min). The obtained amplicons were purified using ExoSap (Vilnius City, Lithuania) and subjected to fluorescent dye‐terminator (ddNTP) sequencing following the manufacturer's recommended protocol for BigDye sequencing chemistry (Applied Biosystems, California, CA, USA) and using the same primers as the PCR. Purified amplicons were sequenced bidirectionally using an ABI 3500 automatic sequencer (Applied Biosystems).

### 2.4 Sequence editing and alignment

The forward and reverse chromatograms were assembled into contigs using Geneious 7.0.6 and edited manually where required. The sequences were aligned in the software MEGA 11 (Tamura et al., 2021) using the Clustal W method (Chenna et al., 2003; Thompson et al., 1994) and subsequently translated into amino acid to verify the presence of premature stop codons or indels. The final matrix was reduced to unique specimen per haplotypes using DAMBE version 7.3.32 (Xia, 2018), a very important criterion to avoid generating unrealistic results (Blair & Bryson, 2017; de Sousa et al., 2021). Our final dataset comprised 547 base pairs and was analysed on the IQ‐TREE WebServer (http://iqtree.cibiv.univie.ac.at/) using its embedded ModelFinder function (Kalyaanamoorthy et al., 2017) to determine the best‐fit evolutionary model (HKY + F + G4).

### 2.5 Phylogenetic analyses

Phylogenetic relationships were inferred using Bayesian inference (BI) approaches in the BEAST version 10.X software (Suchard et al., 2018), with two independent runs of Markov chain Monte‐Carlo (MCMC) with 10 million generations each, and sampling every 1000 generations. A Coalescent Bayesian Skyline was used as a tree prior with an Optimized Relaxed Clock as clock model under a log‐normal distribution. The first 25% of the samples were discarded as burn‐in, and the remaining samples were used to find the maximum clade credibility (MCC) tree and its respective Bayesian posterior probabilities. Convergence and parameter estimates were evaluated using Tracer version 1.7.2 (Rambaut et al., 2018), whereas tree topologies were visualized and examined using FigTree version 1.4.2 (Rambaut & Drummond, 2018). The final matrix of the mitochondrial gene *COI* included 27 terminals of the topotypes as in‐groups and two out‐groups, including three new sequences generated in the scope of this work and additional retrieved data from the GenBank database (Table 1). The out‐group taxa were *Geophagus neambi* Lucinda, Lucena & Assis, 2010, and *G. steindachneri* Eigenmann & Hildebrand, 1910, two species from two groups closely related to the ‘*G*.’ *brasiliensis* species group: *Geophagus* sensu stricto and *G. steindachneri*, respectively (Chuctaya et al., 2022).

**TABLE 1:** The accession numbers of *COI* gene sequences in the GenBank, along with information about the hydrographic basins of the ‘*Geophagus*’ *brasiliensis* species group obtained from Mattos and Costa (2018) and Argolo et al. (2020), the out‐groups obtained from Chakrabarty (2006) and Lima et al. (2024) and the three new sequences generated in the scope of this work (asterisks).

Species	Voucher number	GenBank accession number	Hydrographic basin
‘*Geophagus*’ *brasiliensis*	UFRJ 8365.1	MH538060	Guanabara Bay
‘*G*.’ *brasiliensis*	UFRJ 8365.2	MH538061	Guanabara Bay
‘*G*.’ *brasiliensis*	UFRJ 7738.2	MH538064	Guanabara Bay
‘*Geophagus*’ *diamantinensis*	UFRJ 8245.1	MH538084	Upper Paraguaçu River
‘*G*.’ *diamantinensis*	UFRJ 8245.3	MH538086	Upper Paraguaçu River
‘*G*.’ *diamantinensis*	COLEOAG04	MN295885	Upper Paraguaçu River
‘*Geophagus*’ *iporangensis*	UFRJ 8628.1	MH538065	Ribeira do Iguape River
‘*G*.’ *iporangensis*	UFRJ 8617.1	MH538067	Ribeira do Iguape River
‘*G*.’ *iporangensis*	UFRGS18578	MN295900	Ribeira do Iguape River
‘*Geophagus*’ *itapicuruensis*	UFRJ 9442.3	MH538076	Itapicuru River
‘*G*.’ *itapicuruensis*	UFRJ 9442.4	MH538077	Itapicuru River
‘*G*.’ *itapicuruensis*	UFBA 8591	MN295874	Itapicuru River
‘*Geophagus*’ *multiocellus*	UFRJ 8254.2	MH538079	Contas River
‘*G*.’ *multiocellus*	UFRJ 8254.3	MH538080	Contas River
‘*G*.’ *multiocellus*	UFBA 8596	MN295865	Contas River
‘*Geophagus*’ *obscurus*	UFRJ 9440.1	MH538088	Paraguaçu River
‘*G*.’ *obscurus*	UFRJ 9440.2	MH538089	Paraguaçu River
‘*G*.’ *obscurus*	UFRJ 10026.1	MH538090	Paraguaçu River
‘*Geophagus*’ *pirangaensis*	MZUFV 7654	PV955653*	Upper Doce River
‘*G*.’ *pirangaensis*	MZUFV 13721	PV955654*	Upper Doce River
‘*G*.’ *pirangaensis*	MZUFV 13722	PV955655*	Upper Doce River
‘*Geophagus*’ *rufomarginatus*	URFJ 9518.1	MH538069	Buranhém River
‘*G*.’ *rufomarginatus*	URFJ 1103.1	MH538072	Buranhém River
‘*G*.’ *rufomarginatus*	URFJ 1103.2	MH538073	Buranhém River
‘*Geophagus*’ *santosi*	UFRJ 9998.1	MH538081	Mariana River
‘*G*.’ *santosi*	UFRJ 9998.2	MH538082	Mariana River
‘*G*.’ *santosi*	UFRJ 9998.3	MH538083	Mariana River
*Geophagus neambi*	255_TOC	OR732924	Middle Tocantins River
*Geophagus steindachneri*	UMMZ 243208	DQ119217	Cauca River

### 2.6 Species delimitation

We adopted the principle of integrative taxonomy, which considers the integrated approach between molecular and morphological methods to delimit species (Dayrat, 2005). The Population Aggregation Analysis (PAA), which considers a unique combination of characters to diagnose species, was applied to morphological characters (Davis & Nixon, 1992). The branch coalescence methods General Mixed Yule Coalescent (GMYC single threshold and GMYC multi‐threshold) and the Bayesian Poisson Tree Processes (bPTP) were applied to nucleotide sequences (Zhang et al., 2013). The GMYC considers the time to determine that lineages between the root and transition points in an input ultrametric tree are different species, whereas the bPTP considers the number of nucleotide substitutions to determine that lineages between the root and transition points in an input non‐ultrametric tree are different species (Zhang et al., 2013). The most important premise of these coalescence methods is to include at least two non‐identical haplotypes from each species in the dataset to generate an input phylogenetic tree (Fujisawa & Barraclough, 2013; Monaghan et al., 2009). To understand levels of genetic diversity of the ‘*G*.’ *brasiliensis* species group based on the relative number of nucleotide substitutions, a K2P interspecific genetic distances table was generated using the MEGA 11 software (Tamura et al., 2021).

## 3 RESULTS

### 3.1 Molecular analyses

**FIGURE 1:**
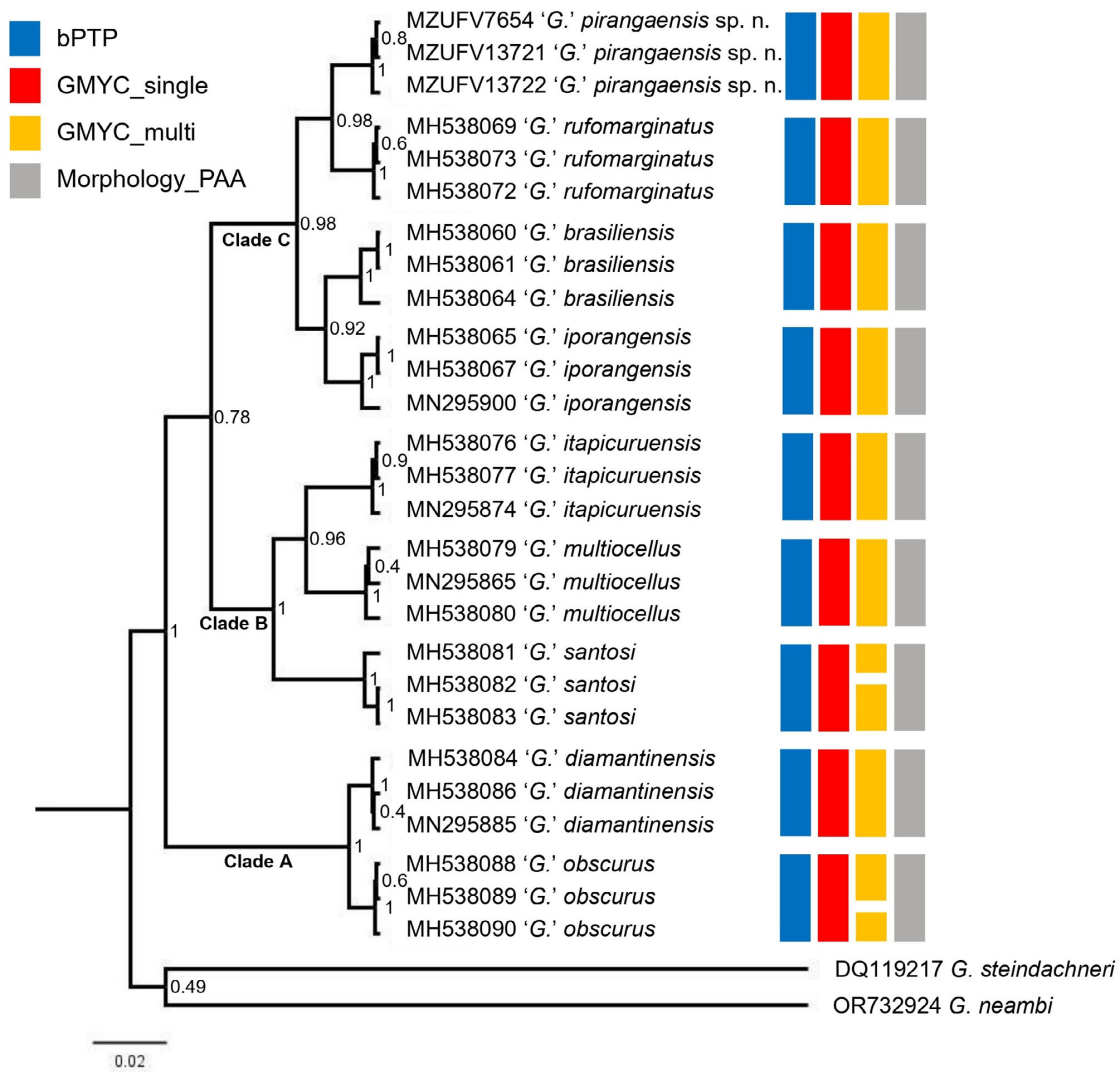
Tree topology estimated by Bayesian inference (BI) showing the phylogenetic relationships among the ‘*Geophagus*’ *brasiliensis* species group, using the mitochondrial gene *COI*. Numbers before terminal species names are voucher numbers. Numbers in front of branches indicate Bayesian posterior probabilities. Coloured vertical bars represent the four species delimitation methods [blue bar: Bayesian Poisson Tree Processes (bPTP); red bar: General Mixed Yule Coalescent (GMYC) single threshold; orange bar: GMYC multi‐threshold; grey bar: Morphology_PAA).

The topology of the BI tree recovered nine well‐supported lineages corresponding to the nominal species of the ‘*G*.’ *brasiliensis* group and the new species here described, grouped into three main clades: the clade A comprising ‘*G*.’ *diamantinensis* and ‘*G*.’ *obscurus*; the clade B comprising ‘*G*.’ *itapicuruensis*, ‘*G*.’ *multiocellus* and ‘*G*.’ *santosi*; and the clade C comprising ‘*G*.’ *brasiliensis*, ‘*G*.’ *iporangensis*, ‘*G*.’ *rufomarginatus* and the new species from the Piranga River basin (Figure 1).

The haplotypes correspond to the new species as a sister group to ‘*G*.’ *rufomarginatus*, with posterior probability greater than 95% (see Figure 1, clade C) and interspecific genetic distance equal to 2.3% (Table 2). The analyses indicated that the haplotypes of ‘*Geophagus*’ from the Piranga River constitute a new species with maximum posterior probability, and the new species was strongly recovered as part of the ‘*G*.’ *brasiliensis* species group (see Figure 1, clade C). The population of ‘*Geophagus*’ from the Piranga River is thus diagnosable from the other species of the group by a unique combination of morphological characters (see Section 3.2.4), by the bPTP, the single‐ and multi‐thresholds of GMYC (see Figure 1) and interspecific genetic distance greater than 2% (see Table 2).

**TABLE 2:** Pair‐wise K2P interspecific genetic distance among species of the ‘*Geophagus*’ *brasiliensis* species group.

	Species	1	2	3	4	5	6	7	8	9
1	‘*Geophagus*’ *brasiliensis*	0.000								
2	‘*Geophagus*’ *diamantinensis*	0.073	0.000							
3	‘*Geophagus*’ *iporangensis*	0.023	0.067	0.000						
4	‘*Geophagus*’ *itapicuruensis*	0.084	0.106	0.073	0.000					
5	‘*Geophagus*’ *multiocellus*	0.065	0.099	0.062	0.043	0.000				
6	‘*Geophagus*’ *obscurus*	0.083	0.015	0.071	0.103	0.096	0.000			
7	‘*Geophagus*’ *pirangaensis*	0.043	0.084	0.039	0.074	0.071	0.085	0.000		
8	‘*Geophagus*’ *rufomarginatus*	0.029	0.085	0.027	0.077	0.064	0.089	0.023	0.000	
9	‘*Geophagus*’ *santosi*	0.065	0.103	0.061	0.056	0.048	0.104	0.057	0.056	0.000

*Note:* The pair‐wise interspecific distances are represented by the average values of the distances calculated for the dataset of species of the ‘*G*.’ *brasiliensis* species group provided in Table 1.

### 3.2 Taxonomic accounts

#### 3.2.1 ‘*Geophagus*’ *pirangaensis* sp. n. Assis, Dergam & Henschel

urn:lsid:zoobank.org:act:BB47CD93‐DB3D‐48A8‐81C1‐A6D74B8BFCDE.

See Figures 2, 3.

**FIGURE 2:**
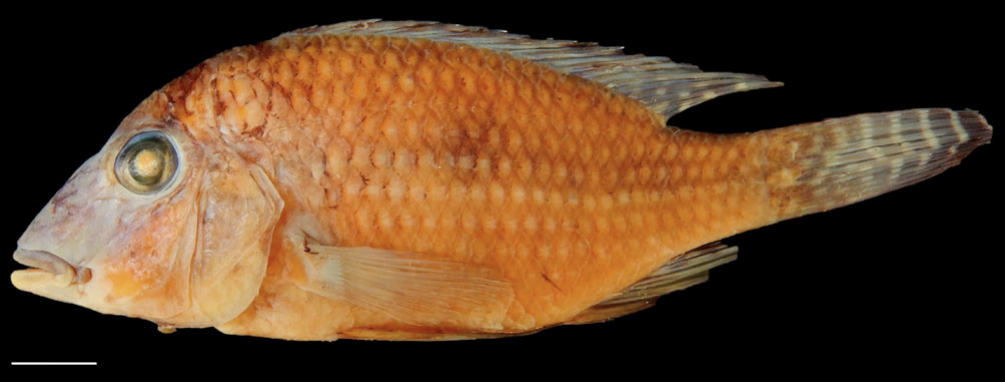
‘*Geophagus*’ *pirangaensis*, MZUFV7654, holotype, 103.89 mm SL, female; Brazil, Minas Gerais, municipality of Ponte Nova: Piranga River, upper Doce basin. Scale bar: 10 mm. Photographed by C. E. de Assis.

**FIGURE 3:**
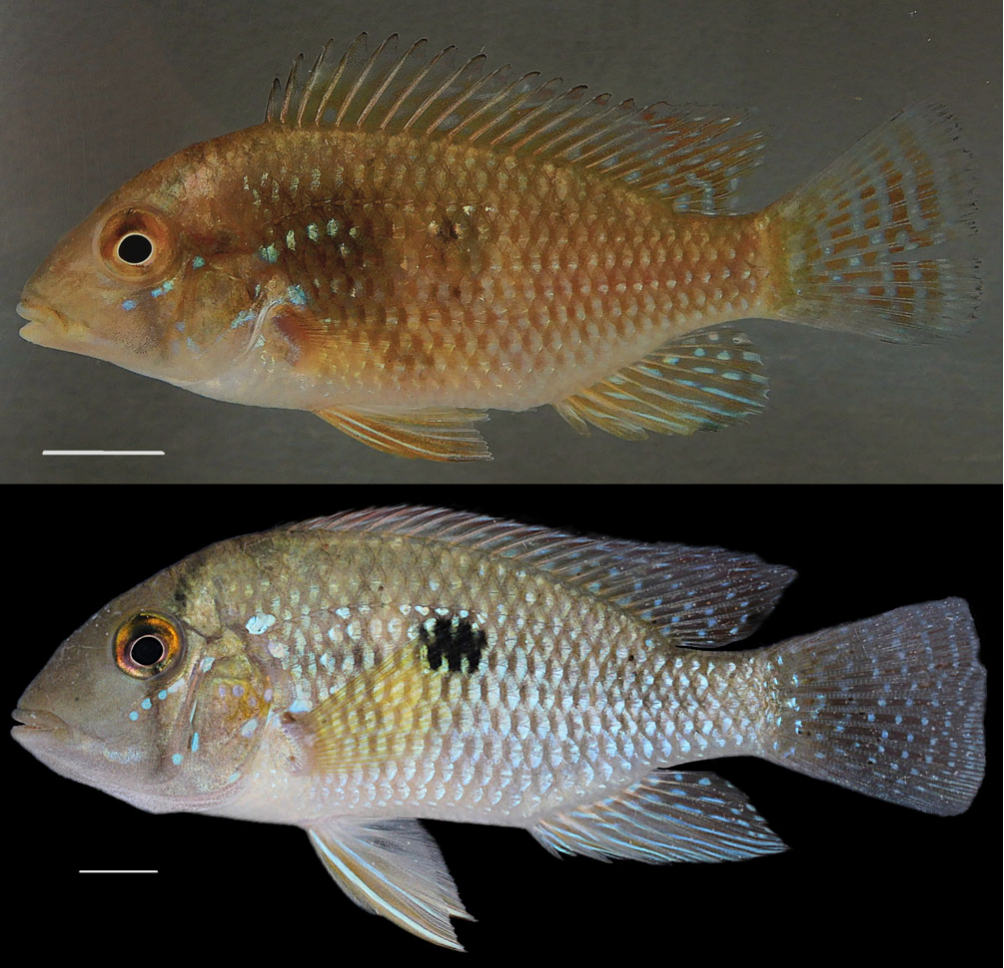
‘*Geophagus*’ *pirangaensis*, new species from Piranga River, upper Doce basin, Ponte Nova municipality, Minas Gerais, Brazil: top, MZUFV13723, live subadult specimen, 63.61 mm SL, collected by E. Henschel and photographed by C.E. de Assis; bottom, MZUFV14666, live adult specimen, 96.21 mm SL, collected by C.E. de Assis and photographed by E. Henschel. Scale bar: 10 mm.

#### 3.2.2 Holotype

MZUFV7654, 103.89 mm SL, female; Brazil, Minas Gerais, municipality of Ponte Nova, Piranga River, 20°25′24.1″S 42°58′05.1″W; collected by F. Fernandes, 16 September 2019.

#### 3.2.3 Paratypes

All from Brazil. MZUFV7766 (2), 107.87–109.71 mm SL; same locality of holotype; collected by F. Fernandes, 5 March 2020. MZUFV8217 (4) 81.68–117.92 mm SL; same locality of holotype; collected by C. E. de Assis, 11 September 2020. MZUFV11099 (4), 88.91–122.51 mm SL; same locality of holotype; collected by F. Fernandes, 9 August 2022. MZUFV13721 (1), 105.45 mm SL; collected with holotype. MZUFV13722 (1), 101.43 mm SL; collected with holotype. UFMG‐ICT 4031 (2), 83.92–86.65 mm SL; same locality of holotype; collected by F. Fernandes, 9 August 2022. UFMG‐ICT 4032 (2), 83.49–88.64 mm SL; same locality of holotype; collected by C. E. de Assis, 11 September 2020.

#### 3.2.4 Diagnosis

‘*G*.’ *pirangaensis* possess a unique diagnostic feature within the ‘*G*.’ *brasiliensis* group characterized by the presence of clear, rounded spots in the posteriormost dorsal‐fin rays of fixed adult specimens (vs. absence, or, when present, spots are elliptical). It also differs from ‘*G*.’ *brasiliensis* by having a considerably longer snout (46.22%–52.74% HL vs. 38.36% HL) and terminal mouth (vs. subdorsal); from ‘*G*.’ *diamantinensis* by having a taller head (94.08%–101.15% HL vs. 77–86% HL) and terminal mouth (vs. subterminal); from ‘*G*.’ *iporangensis* by having a caudal fin with a rounded distal margin (vs. straight distal caudal‐fin margin) and 25 or 26 longitudinal scales in the E0 series (vs. 27 or 28); from ‘*G*.’ *itapicuruensis* by having a dark and rounded mid‐lateral spot of live specimens (vs. dark and vertically elliptical mid‐lateral spot) and 14 or 15 spines on the dorsal fin (vs. 13); from ‘*G*.’ *multiocellus* by the absence of small bright spots in the central region of the bluish spots on the caudal fin of living specimens (vs. presence) and terminal mouth (vs. subterminal); from ‘*G*.’ *obscurus* by having small bluish spots in the opercular region of living specimens (vs. large bluish spots in the opercular region); from ‘*G*.’ *rufomarginatus* by the absence of bluish dots on the most anterior soft rays of the anal fin of living specimens (vs. presence), presence of denticles on gill rakers of the first branchial arch (vs. absence) and terminal mouth (vs. subterminal); and from ‘*G*.’ *santosi* by the absence of bluish and longitudinal lines in the basal region of the caudal fin of living specimens (vs. presence) and terminal mouth (vs. subterminal).

#### 3.2.5 Description

Morphometric measurements are shown in Table 3. Small species, largest specimen examined measured 122.64 mm SL. Body moderately narrow and laterally compressed. Dorsal profile of head nearly straight from tip of snout to posterior margin of eye, convex from that point to dorsal‐fin origin. Ventral profile of body slightly convex from mouth to pelvic‐fin origin, markedly convex from that point to anal‐fin posterior margin, straight from point to end of caudal peduncle.

**TABLE 3:** The morphometric measurements of ‘*Geophagus*’ *pirangaensis*.

	H	Range (*n* = 16)	Mean	SD
Standard length (mm)	103.89	81.68–122.64	‐	‐
Percentage of standard length				
Body depth	40.98	39.39–43.92	40.89	1.51
Predorsal length	42.65	39.45–44.84	42.51	1.52
Dorsal‐fin base length	54.36	54.36–58.11	56.55	1.29
Last dorsal‐fin spine length	15.82	12.41–15.82	14.41	0.98
Prepelvic length	40.34	38.85–43.25	40.31	1.29
Pelvic‐fin length	33.15	26.62–33.15	29.51	2.63
Pelvic‐fin spine length	15.69	12.69–15.77	13.89	1.25
Pectoral‐fin length	29.83	26.65–31.72	29.27	1.61
Anal‐fin base length	18.01	17.19–19.92	18.46	0.81
Last anal‐fin spine length	15.93	12.95–15.93	14.25	0.96
Caudal peduncle length	15.55	10.67–15.55	13.08	1.29
Caudal peduncle depth	14.06	13.36–15.12	14.46	0.52
Head length	35.28	33.42–36.24	35.27	0.82
Percentage of head length				
Snout length	50.51	46.22–52.74	49.85	1.84
Preorbital depth	46.17	41.88–48.44	46.10	1.77
Head width	45.59	44.14–51.76	47.47	2.56
Head depth	94.07	90.03–101.15	96.41	3.35
Orbital diameter	23.79	21.74–26.43	23.81	1.45
Interorbital width	30.89	28.81–35.02	31.02	1.84
Upper jaw length	27.26	25.12–30.52	27.01	1.49
Lower jaw length	28.57	24.08–28.57	26.46	1.51

*Note:* The holotype measurements are included in the range.

Abbreviations: H, holotype; SD, standard deviation.

Snout moderately pointed; nostrils located between tip of snout and anterior orbital margin. Mouth terminal, distal end of maxilla in a vertical line not reaching the anterior margin of the eye. Lower and upper jaw at same level. Eyes closer to dorsal profile of head. No serrations on operculum.

Insertion of first spine of dorsal fin in a vertical line slightly posterior to posterior margin of operculum. Tip of dorsal fin pointed, not surpassing median region of caudal fin. Tip of anal fin pointed, not surpassing median region of caudal fin. Caudal fin subtruncated, with posterior margin rounded. Pectoral fin trapezoidal, with posterior margin rounded, distal margin not exceeding posterior margin of mediolateral spot. Pelvic fin pointed, distal margin not surpassing insertion of third spine of anal fin. Insertion of first spine of anal fin in a vertical line through region between 13th and 14th dorsal‐fin spines. Dorsal‐fin elements: XIV or XV + 10 or 11 (16); anal‐fin elements: III + 7 or 8 (16); pectoral‐fin rays: 13 or 14 (16); pelvic‐fin elements: I + 5 (16); caudal‐fin rays: iv or v + 15 or 16 + iii or iv (16).

Sides of head covered by cycloid scales below eyes. Snout and ventral region of head devoid of scales. Pectoral region, trunk and caudal peduncle covered by ctenoid scales. Head and pectoral scales smaller than scales on flank. Two small scales at base of posteriormost dorsal‐fin rays. Anal fin devoid of scales. Approximately first third of caudal fin covered by tiny scales, smaller than caudal peduncle scales. Two series of scales between upper and lower branches of lateral line. Longitudinal series of scales between upper branch of lateral line and dorsal‐fin origin: 4 or 5; longitudinal series of scales between lower branch of lateral line and anal‐fin origin: 5 or 6; scales in longitudinal series E0: 25 or 26; scales in longitudinal series E1: 26 or 27; scales in longitudinal series E2: 25 or 26; scale series on cheek: 4 or 5; scales on upper branch of lateral line: 18 or 19; scales on lower branch of lateral line: 11 to 13; longitudinal series of scales on caudal peduncle: 16 or 17.

Premaxillary teeth conical and slightly curved posteriorly; teeth of outer series enlarged in region of symphysis. Shape and arrangement of dentary teeth similar to premaxillary teeth, but slightly smaller. One supraneural. Five rays in branchiostegal membranes. Fleshy lobe on upper part of first branchial arch. Denticles on gill rakers of first branchial arch. Number of gill rakers on first branchial arch: 8 or 9 in ceratobranchial; 1 in articulation; 7 or 8 in epibranchial. Ceratobranchial gill rakers short and blunt. Number of dorsal fin proximal radials: 25 + 1; number of anal‐fin radials: 9 + 1. Number of pleural ribs: 11; number of epipleural ribs: 13; number of vertebrae: 14 + 14.

#### 3.2.6 Colouration in alcohol

Ground colour of body varies from light brown to dark brown, with some dark chromatophores concentrated in predorsal region; ventral region of body slightly lighter than dorsal region, generally in shades of light beige. Head slightly darker than body, especially in preorbital region. Eyes brown with dark upper margin. Dark, vertical bars on sides of body (inconspicuous in some specimens), first above pectoral‐fin origin and last close to base of caudal fin. Dark, round spot on middle of body. Dark, slightly inclined stripe usually present in infraorbital region, extending from lower orbital margin to angle of preoperculum. Dorsal‐fin colouration varies from light brown to dark brown, with several light, rounded punctuations in posterior region. Anal‐fin colouration varies from light brown to dark brown, with light punctuations on last three soft rays. Pectoral fin entirely light brown. Pelvic fin almost entirely light brown, with first spinous ray darkest. Caudal fin light brown, with five to seven dark, transverse bars, alternating with light transverse stripes and more evident in partially retracted rays.

#### 3.2.7 Colouration in life

Sides of body light grey in adult specimens and light brow in subadult specimens, with inconspicuous darker transverse bars. Bluish spots on body scales, forming longitudinal series that extend from operculum to caudal peduncle, larger and more evident posteriorly in adult specimens. Pectoral and ventral regions lighter than rest of body. Head light grey in adult specimens and light brow in subadult specimens and darker in preorbital region. Orange iris, with dark upper and lower margins. Bluish dots in infraorbital region, preoperculum and operculum, generally organized in oblique series just below the eyes and more scattered in the region of preoperculum and operculum. Dark mid‐lateral spot conspicuous in adult specimens and inconspicuous in subadult specimens. Dark, slightly inclined stripe present in infraorbital region, extending from lower orbital margin to angle of preoperculum. Dorsal fin dark grey in adult specimens and light brown in subadult specimens, with several clear and conspicuous bluish dots in most posterior region. Anal fin same as dorsal fin, but with bluish stripes parallel to anterior rays and bluish dots on last three soft rays. Pectoral fin yellowish in adult specimens and light brown in subadult specimens. Pelvic fin light grey in adult specimens and light brown in subadult specimens, and with bluish stripes parallel to outermost rays. Caudal fin dark brown, and with five to seven bars alternating with bluish transverse stripes in posterior region and region and bluish dots on median region.

#### 3.2.8 Geographical distribution

**FIGURE 4:**
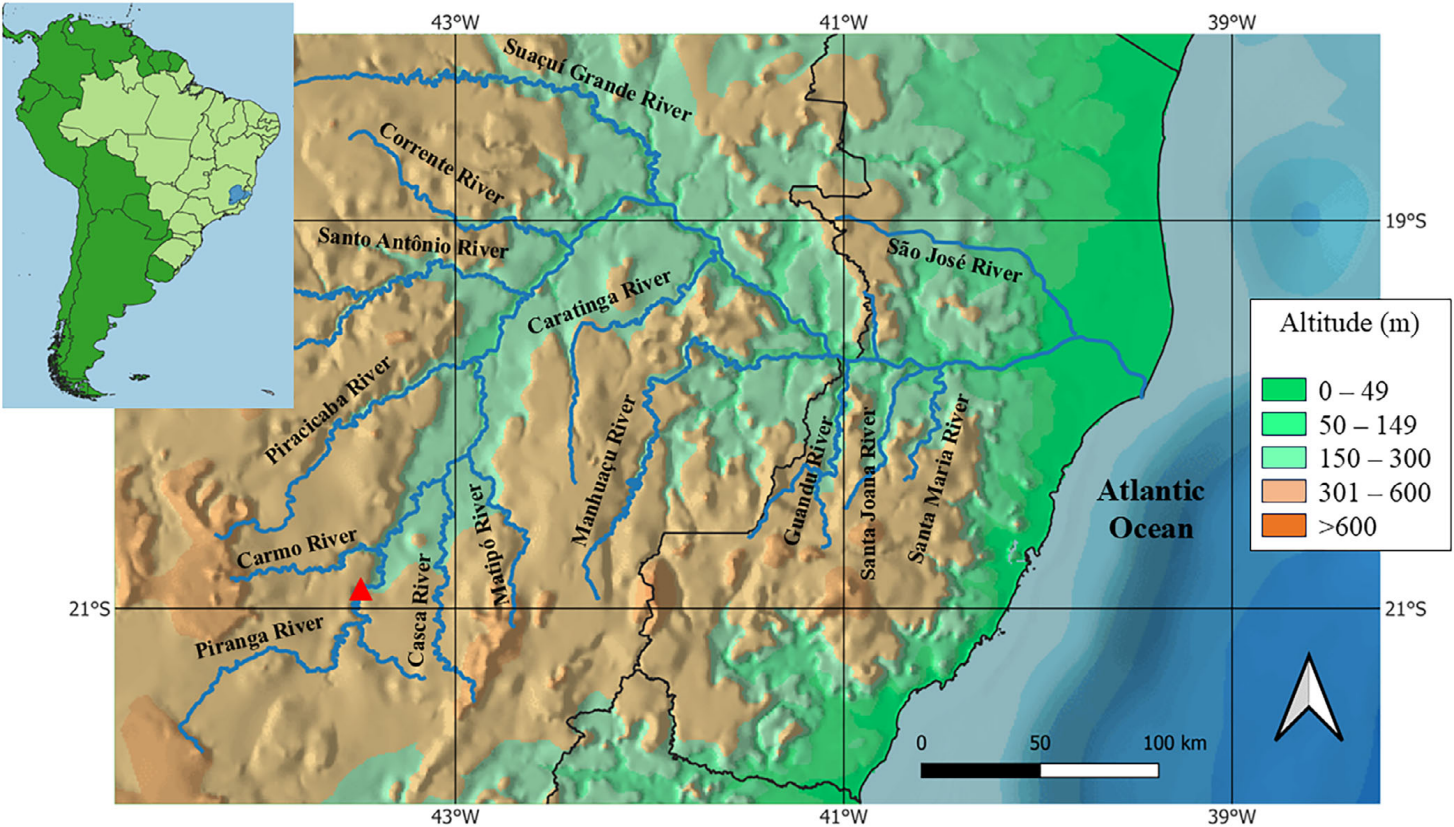
Type locality of ‘*Geophagus*’ *pirangaensis* in the Doce River basin (red triangle).

The new species is currently known only from its type locality in the middle course of the Piranga River, in the municipality of Ponte Nova, Minas Gerais, Brazil (Figure 4).

#### 3.2.9 Etymology

The specific epithet ‘*pirangaensis*’ is a noun referring to the Piranga River, a tributary of the Doce River, where the known populations of the species were collected.

#### 3.2.10 Habitat notes

**FIGURE 5:**
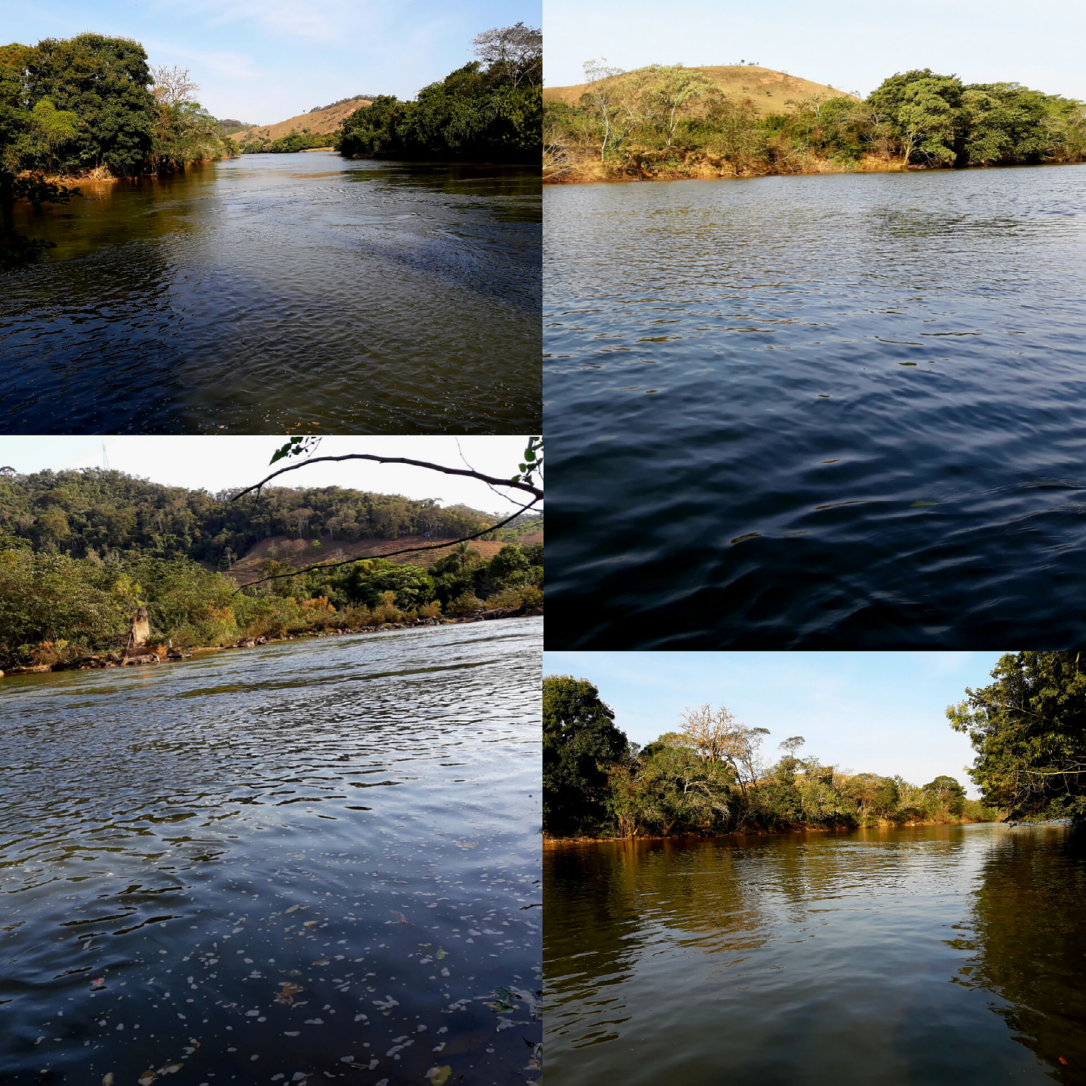
Middle course of the Piranga River in the municipality of Ponte Nova, state of Minas Gerais, where the type series of ‘*Geophagus*’ *pirangaensis* was collected. Photographed by C.E. de Assis.

The type locality is characterized by the presence of backwaters and abundant water in shallower areas. Sandbanks are covered by trees and shrubs of secondary forest. The width of the river varies between 50 and 70 m, with abundant riparian forest, and the riverbed is not covered by vegetation (Figure 5). The river substrate is muddy and sandy and partially covered by a few rocks.

## 4 DISCUSSION

The ‘*G*.’ *brasiliensis* species group is a monophyletic lineage first proposed by Kullander (1998), considering the phylogenetic relationships and systematics of South American cichlids, being supported by other subsequent studies using analyses of mitochondrial and nuclear genes (Chuctaya et al., 2022; López‐Fernández et al. 2005,b; López‐Fernández et al., 2010). Until now, this group has been represented by eight species from eastern South America (Argolo et al., 2020), with populations from northeastern Brazil being the best studied using integrative approaches (Mattos & Costa, 2018). However, populations from southeastern Brazil lacked comprehensive integrative studies.

Integrative taxonomy is a multidisciplinary approach that combines morphological and molecular evidence as the main tools for delineating new taxa (Dayrat, 2005). This concept presents a more robust dataset and has shown many advances in studies of the subfamily Cichlinae, mainly the description of new taxa at the species level that were previously considered as a single species or even a complex of cryptic species (Lozano et al., 2022; Mattos et al., 2015; Mattos & Costa, 2018; Říčan et al., 2019; Ximenes et al., 2021). The application of molecular methods to delineate species is the main tool that allows the detection of the great diversity of lineages and species that could not be detected using only traditional morphological methods. For example, studies with populations of the genus *Australoheros* from coastal basins of southeastern Brazil have revealed several species with unique molecular diagnostic characters (Ottoni et al., 2019; Silva et al., 2023). Integrative taxonomy allows for the precise recognition of cryptic species, and advances in this approach will be fundamental to addressing the ‘Freshwater Biodiversity Crisis’, a global phenomenon referring to the decline and threat of extinction of aquatic species (Albert et al., 2020; Ottoni et al., 2025). Fish species not previously recognized by science or that go unnoticed are often left out of conservation measures, and they urgently require conservation intervention (Ottoni et al., 2023, 2025; Tickner et al., 2020; Sayer et al., 2025). The new species described in this study is part of a complex of cryptic species and was previously considered a subpopulation of ‘*G*.’ *brasiliensis*, going unnoticed during the threat assessment criteria established by the International Union for Conservation of Nature (IUCN). From now on, populations of this new taxon, endemic to the Rio Doce, can be considered in the planning of conservation measures for the species.

Multiple lines of taxonomic evidence have greatly expanded the descriptions of new species of Neotropical fish, whereas several studies using mitochondrial genes *COI* and *Cytb* have been applied to understand the phylogenetic relationships of the Cichlidae family (Chuctaya et al., 2022; Kullander et al., 2010; Ottoni & Mattos, 2015; Varella et al., 2023). The phylogenetic relationships of the species within ‘*G*.’ *brasiliensis* group inferred in this study were based on the mitochondrial gene *COI* and recovered this group as a well‐supported clade (see Figure 1), consistent with the phylogeny proposed by Chuctaya et al. (2022). The new species was recovered as a sister group to ‘*G*.’ *rufomarginatus* (posterior probability = 0.98) in the BI phylogeny tree (see Figure 1, clade C) and with an interspecific genetic distance equal to 2.3% (see Table 2), exceeding the 2% threshold values for the *COI* gene (Ward, 2009). A sister group relationship between species occurring in close hydrographic regions can be explained by biogeographic events that occurred during the formation of coastal paleodrainages in eastern Brazil, for example, the marine incursions and regressions during the ice ages (Pio & Carvalho, 2021; Thomaz et al., 2015; Thomaz & Knowles, 2018). In fact, similar phylogenetic relationships have been observed in populations of other fish species that also inhabit these coastal basins such as *Hypomasticus copelandii* (Steindachner, 1875), *Hoplias* aff. *malabaricus* (Bloch, 1794), *Megaleporinus conirostris* (Steindachner, 1875) and *Oligosarcus acutirostris* Menezes, 1990 (Mendes et al., 2022; Pereira et al., 2013; Ramirez et al., 2017; Santos et al., 2009; Wendt et al., 2019). Despite this, our results indicate that populations of the Buranhém River are, indeed, ‘*G*.’ *rufomarginatus*, and that populations from the Doce River represent a new endemic species, with a level of endemism similar to that observed in other fish species of the basin, such as *Trichomycterus* spp. and *Deuterodon pedri* Eigenmann, 1908 (Costa et al., 2023; Reis & de Pinna, 2022; Silva et al., 2017).

It has been almost 8 years since the last description of a new species within the ‘*G*.’ *brasiliensis* species group. The last four species were described using an integrated approach with morphological characters and mitochondrial genes (*COI* and *Cytb*), indicating a progress in the taxonomy of this species group (Mattos et al., 2015; Mattos & Costa, 2018). Mattos et al. (2015) described ‘*G*.’ *diamantinensis*, an endemic species from the Paraguaçu River in Chapada Diamantina, Bahia, which differs from ‘*G*.’ *pirangaensis* by having a lower head (77%–86% HL vs. 94.08%–101.15%) and a subterminal mouth (vs. terminal). Later, Mattos and Costa (2018) described three new species of the group from coastal basins in the Bahia state: ‘*G*.’ *multiocellus*, ‘*G*.’ *rufomarginatus* and ‘*G*.’ *santosi*, from the Contas, Buranhém and Mariana rivers, respectively. After the photographs of the holotype of these three species available in Mattos and Costa (2018) were analysed, it was possible to observe that ‘*G*.’ *pirangaensis* can be distinguished from the three mainly by the position of the mouth and in the colour patterns of the caudal and anal fins (see Section 3.2.4).

The knowledge about the ichthyofauna of the Piranga River sub‐basin is relatively scarce; although it has been recognized as one of the regions of ichthyofaunal endemism in the Doce River basin (Sarmento‐Soares et al., 2022; Vieira‐Guimarães et al., 2024), major taxonomic work has been restricted to the Trichomycteridae family (Costa et al., 2023; Reis & de Pinna, 2022). Several collecting expeditions carried out over the past 25 years have revealed only two new fish species from the Piranga River: *Characidium krenak* Oliveira‐Silva et al., 2022, and *Neoplecostomus pirangaensis* Oliveira & Oyakawa, 2019 (Oliveira & Oyakawa, 2019; Oliveira‐Silva et al., 2022). In this study, a new species is described to implement taxonomic studies involving the ichthyofauna of this sub‐basin. The new species ‘*G*.’ *pirangaensis* was recorded in the middle course of the Piranga River, and its geographic distribution is restricted; thus, it is considered endemic to this region (see Figure 4).

Two nominal species of the ‘*G*.’ *brasiliensis* species group have been reported from the Doce River basin: ‘*G*.’ *brasiliensis* and ‘*G*.’ *rufomarginatus*. The first of these is the result of studies by Haseman (1911), who described a subspecies of ‘*G*.’ *brasiliensis* (‘*G*.’ *brasiliensis iporangensis*) from the Ribeira do Iguape River based on morphological characters. The comparative material used in Haseman's (1911) study included specimens from the Doce River in Minas Gerais that were considered as ‘*G*.’ *brasiliensis*. However, the morphological characters considered by Haseman (1911) were not very comprehensive and non‐informative for acknowledging the identity of these specimens. The second species was described by Argolo et al. (2020), who applied species delimitation methods based on morphological characters and sequences of the mitochondrial gene *COI* obtained from specimens from the upper and middle Doce River in Minas Gerais, as well as from the Buranhém River in Bahia. Using geometric morphometric data and the GMYC, multi‐rate Poisson Tree Processes (mPTP) and Automatic Barcode Gap Discovery (ABGD) methods, Argolo et al. (2020) expanded the geographic distribution of ‘*G*.’ *rufomarginatus* to the upper and middle Doce River basin. This species was originally known to be endemic to the Buranhém River (Mattos & Costa, 2018). It is worth noting that the comparative material analysed in this study are the topotypes of each of the eight species of the ‘*G*.’ *brasiliensis* species group, among which the haplotypes of ‘*G*.’ *pirangaensis* were distinguished from those of ‘*G*.’ *rufomarginatus* according to the four species delimitation methods (see Figure 1 and Section 3.2.4). However, there is still controversy about the true identity of ‘*G*.’ *rufomarginatus* in the Doce River basin (Argolo et al., 2020), and thus, this species deserves a formal taxonomic review, which goes beyond the scope of this study. The results of this study show that ‘*G*.’ *pirangaensis* is a new species, delimited by three species discovery methods and characterized by a combination of morphometric and meristic traits that distinguish it from the two previously mentioned species. Furthermore, this study highlights the importance of integrative approaches in the taxonomy of the ‘*G*.’ *brasiliensis* group, helping to clarify fish diversity in threatened areas with high levels of endemism.

## 5 COMPARATIVE MATERIAL EXAMINED

All from Brazil. ‘***G*.’ *brasiliensis***: MNHN A‐9503, holotype (photograph), 102 mm SL, near Rio de Janeiro, Guanabara Bay drainages. ***Chromys unimaculata***: MNHN A‐9506, holotype, (photograph), 126 mm SL, near Rio de Janeiro. ‘***G*.’ *diamantinensis***: UFRJ8833, holotype (photograph), 68.5 mm SL, Bahia, Andaraí, Paraguaçu River. ‘***G*.’ *iporangensis***: FMNH54202, holotype (photograph), 87 mm SL, São Paulo, Iporanga, Ribeira do Iguape River. ‘***G*.’ *itapicuruensis***: FMNH54365, holotype (photograph), 113 mm SL, Bahia, Queimados, Itapicuru River. ‘***G*.’ *multiocellus***: UFRJ11764, holotype (photograph), 101.4 mm SL, Bahia, Iguaí, Cambiriba River, Contas River basin. ‘***G*.’ *obscurus***: MNHN9511, syntypes (photograph) (2), 111–118 mm SL, Bahia, Salvador, Paraguaçu River; UFRJ10026, topotype (photograph), 101.5 mm SL, Bahia, Lençóis, Paraguaçu River. ‘***G*.’ *rufomarginatus***: UFRJ9994, holotype (photograph), 96.8 mm SL, Bahia, Porto Seguro, Buranhém River. ‘***G*.’ *santosi***: UFRJ11765, holotype (photograph), 110.6 mm SL, Bahia, Ituberá, Mariana River.

## AUTHOR CONTRIBUTIONS

Cidimar E. de Assis: study design, specimen collection, data collection, morphological data analysis, figure preparation, manuscript writing and manuscript editing. Jorge A. Dergam: specimen collection, manuscript revision and supervision. Amanda F. Cunha: manuscript revision and supervision. Valéria N. Machado: DNA extraction, DNA sequencing and manuscript revision. Tomas Hrbek: DNA sequencing and manuscript revision. Natállia M. de F. Vicente: DNA extraction and manuscript revision. Victor de Queiroz: specimen collection and manuscript revision. Elisabeth Henschel: study design, molecular data analysis, specimen collection, supervision and manuscript revision.

## FUNDING INFORMATION

Fundação Espírito‐santense de Tecnologia ‐FEST, grant numbers: 101103/2022.

## Data Availability

The data that support the findings of this study are available from the corresponding author upon reasonable request.
